# The Impact of the Excellence Program on Employee Performance at Aseer Central Hospital in Saudi Arabia: A Cross-Sectional Study

**DOI:** 10.7759/cureus.32862

**Published:** 2022-12-23

**Authors:** Abdullah Saeed, Abdullah AlShafea, Maliha Nasser, Abdulrahman Alasmari

**Affiliations:** 1 Action Research, Ministry of Health, Abha, SAU; 2 Resarch, Ministry of Health, Abha, SAU; 3 Human Resources, Ministry of Health, Abha, SAU; 4 Planning and Transformation, Ministry of Health, Abha, SAU

**Keywords:** reward, encourage, quality improvement, employee satisfaction, excellence

## Abstract

Introduction

Motivation is a power that directs employees toward achieving their special goals and the organization's objectives in general. Recently, motivation has been considered one of the most important issues in the workplace since each organization wants to get the most out of its resources, including its manpower. In Saudi Arabia, the Aseer Health Directorate like the other regions, has a monthly reward containing certification and online announcements of employee excellence via email and website. The annual ministry program has three levels: 10% in salary for employees who have received excellence certification from the general director, 20% if they add research, and 30% if they register a patent.

Methods

A cross-sectional study was conducted in the Aseer Central Hospital among healthcare providers (HCP) of all categories using structured interviews with three parts: socio-demographic, knowledge about criteria for excellence in the program, and opinion toward the program and its effects. The sample size was calculated using the total number of employees in the hospital, with 77 being the bare minimum required. The sampling method used was systematic random sampling with a list of statistical analyses from the SPSS statistical package (IBM Corp., Armonk, NY, USA). The statistical analysis description for all variables was based on frequency and presence, followed by bivariate analysis.

Results

In the study, 103 participants who were physicians made up 28.2% of the sample, followed by nurses at 32%, and other health professionals at 39.8%. Males made up 64.1% and females 35.9%. While 55.3% of individuals claimed to be aware of the standards of excellence allowance, 44.7% of participants denied doing so. Around 7.8% of employees missed work or arrived late, compared to 92.2% who didn't, and 75% of those who had proof in their files received a pay cut or other penalty. The average employee's age was 33.5 years old, and 4.05% of them believed they performed better than their coworkers. An average of 4.43 employees believe that there will be more competition when the number of allowances increases. While the excellence allowance has a favorable effect as indicated by the 4.55 mean. With a mean of 2.47 and 2.45, the majority of employees, respectively, did not agree with the transparent and fair selection process.

Conclusion

The purpose of the study was to demonstrate the significance of rewarding and encouraging employees, as well as the positive impact of this system on increasing their performance and productivity in order to implement the health organization's strategy and improve healthcare delivery and quality. There is a high percentage of employees who are dissatisfied with the transparency and fairness of the selection criteria, and some of them believe that team performance should be improved rather than individual performance. So the recommendation is to improve the selection criteria, make them more specific and transparent, and increase the number of teams and individuals who can be enrolled to improve the healthcare delivery system.

## Introduction

Motivation is a power that directs employees toward achieving their special goals and an organization's objectives. Recently, it is considered one of the most important issues in the workplace since each organization wants to get the most out of its resources, including manpower [1]. The Saudi Ministry of Health has set the excellence allowance for its Saudi employees in their various specialties and job categories at no more than 30% of the salary of the employee's first-degree level. It aims to honor its outstanding employees and motivate all of its efforts to change the work culture to achieve better work quality and improvement [2].

The excellence allowance has regulations and requirements that have to be fulfilled by the employee to be eligible to receive it. These requirements are mentioned in the guide to financial rights and benefits for Saudi healthcare practitioners. It stated that the allowance is to be given to the healthcare provider (HCP) in accordance with mandatory regulations, including a basic element of getting "excellent" in the final staff appraisal of the last two years. The nominee has to be disciplined and productive, and no punitive actions should have been taken against him or her over the past three years. In addition to this basic element, the HCP has to achieve one of the following elements to get a 10%/20%/30% allowance: receive awards or honors from an accredited organization; participate in volunteer work to serve the community; engage with worksheets in medical conferences and symposia, submit scientific research and experiments; author a scientific or educational book; or publish in peer-reviewed journals. Also, obtaining a patent for a medical device or developing a new technique that participates in medical development is allowed, and the given allowance here is 30% [3].

The presence of motivated healthcare workers affects the health system's ability to provide high-quality medical care. Motivation has been linked with many benefits, including improved performance, lower job burnout, stronger retention, and lower levels of staff turnover [4]. The best healthcare companies today have a reputation for performance excellence, which is necessary to deliver high-quality care and services [5]. Although most healthcare interventions are successful and effective, a large number of efforts focus on eliciting learning from mistakes, while the possibilities of learning can be widened to include learning from excellence to enhance learning in several ways, like intrinsic motivation [6]. The motivators of employees that participate in target settings can be broadly divided into intrinsic and extrinsic motivators. Intrinsic motivators are initiated from within the employee and are an end in themselves, where the employee is the main beneficiary; extrinsic motivators are controlled by the employer and perceived as a means to an end, where the employer is the main beneficiary [7].

Motivation can be an outcome of the interaction of multiple emotional and tangible elements such as the feeling of need, incentive, or reward [8]. Employees are motivated in multiple ways, including through trust in the employer, good managers, job security, being appreciated, being encouraged, peer motivation, and money and benefits. Praise and constructive criticism are considered the main motivators, and incentive programs are usually based on them but are very tricky. Making employees focus on their results is a major motivator when they are well-trained and the outcomes of their job are assessed and contrasted with a standard or objective [9]. The majority of employees need motivation to feel better about their jobs and work perfectly. Some are financially motivated, while others find that recognition and rewards are personally motivating. Others can be motivated by the feeling of accomplishment and achievement of their personal and professional goals, and those are usually less affected by incentives or rewards [2].

Most employers still work with systems that do not support financial stimuli for the employee, which is in contrast to the fact that most employees are challenged to increase their productivity and are reinforced with financial rewards [10]. From a motivational point of view, the financial benefits reward category has been rated most highly by employees [11]. Creating a reward system that is based on performance is one of the greatest methods to improve organizational performance, which is the main concern for each organization [12]. Employees tend to gain motivation for a defined intent, while organizations focus on performance growth and enhanced efficiency by rewarding employees [13]. Teamwork is an essential part of health care, and significant advantages can be obtained from the presence of teams. It has been suggested that using team-based rewards might improve teamwork [14].

The use of team incentives is a common strategy used to encourage team performance. But a study demonstrated that this approach sometimes gives the opposite results because some team members perceive it as inequitable [15]. A study done in Pakistan in 2014 found that there is a strong and positive relationship between employee motivation and performance [1]. In a study done in Port Said, Egypt, in 2021, nurses assessed their job performance as low and attributed this to the fact that they felt frustration and lacked motivation due to the low level of Human Resources Practice (HRP) submitted to them [16]. Another cross-sectional study done in China in 2019 used the effort-reward imbalance (ERI) model to explore associations between ERI and burnout among healthcare professionals in healthcare centers [17]. The Canada Awards for Excellence have been established and awarded annually in four categories, including the Award of Excellence for Invention and Research. It honors those who have made excellent contributions to research or innovation, solved a problem, or effectively taken advantage of a chance that improved search and rescue for the benefit of response and/or prevention [18]. This study was conducted to evaluate the HCPs' knowledge of the excellence allowance and its requirements, rules, and criteria at Aseer Central Hospital.

## Materials and methods

Method

A descriptive cross-sectional study was conducted at Aseer Central Hospital, Abha, Saudi Arabia. The ethical approval was granted by the Aseer Institutional Review Board (approval no. REC12-08-2022). The sample size was calculated based on the total number of employees at Aseer Central Hospital (2100 HCPs). Taking into account the population size, we used 5% as the population probability, which is the maximum allowed to get an excellence allowance by the Ministry of Health. The minimum sample size required to achieve 95% confidence was 71 employees; the selection of participants was made using systematic random sampling from the department. The study was conducted between September 1st to October 31st, 2022. The study's participants were male and female HCPs. Administrative jobs were excluded because they have different excellence criteria and company workers are not paid by the Ministry of Health.

Structured questionnaires were distributed to the participants. These featured sociodemographic factors (age and gender) and variables based on criteria for the excellence program from the Saudi Ministry of Health, such as no history of absence, excellent performance evaluation in the last two years, participation in patent or research, no vacation other than annual leave and sick leave, the challenges and drawbacks of the current program, and the opinion of HCPs towards improvement. All variables in the study were self-reported.

The questionnaire used the five-point Likert scale (strongly disagree, disagree, neutral, agree, and strongly agree) and the responses were coded from 1 for strongly disagree through 5 for strongly agree. The descriptive analysis includes first the frequency and percentage for categorical variables and the mean standard deviation for continuous variables. Second, the mean score for each group category. The t-test with a mean score for the question "Do you see your job performance better than your colleague?" compared with the responses to the remaining variables had a significance level of 0.05. The data were analyzed by SPSS (IBM Corp., Armonk, NY, USA).

## Results

There was no missed response from the 103 answers. Table 1 shows physicians represented 28.2% (29) of the sample while 32% (33) were nurses, and the remaining 39.8% (41) were other health practitioners. Of the total participants, 64.1% (66) were male and 35.9% (37) were female. Around 44.7% (46) of the participants denied knowing the standards of excellence allowance while 55.3% (57) reported they were aware. About 92.2% (95) had no absence or didn't arrive at work late while 7.8% (8) did, with 75% (6) of them having documentation in their files on salary-cut or punishment. When asked about any participation in previous publishing or patent, 68.9% (71) answered negatively while 31.1% (32) revealed they had participated. Regarding favoritism, 14.6% (15) were seeing there is no favoritism in the nomination versus 85.4% (88) stating there is, although 35.9% (37) of the participants have been elected to get the allowance. Around 78.6% (81) had been elected for the monthly excellence certificate whereas 92.2% (95) are considering it a motivation to give more. About 96.1% (99) got an excellent degree in their evaluations for the last two years. By asking about team excellence, if it is fairer, 59.2% (61) agreed that it is fair while the rest did not.

**Table 1:** Descriptive analysis of study variables

Variable	Frequency	Percentage
Specialty		
Physician	29	28.2%
Nurse	33	32.0%
Pharmacists	9	8.7%
Other	32	31.1%
Gender		
Male	66	61.1%
Female	37	35.9%
Do you have knowledge of the criteria for excellence allowance?		
Yes	57	55.3%
No	46	44.7%
Have you ever been absent or delayed without an excuse?		
Yes	8	7.8%
No	95	92.2%
Do you have in your record registered absences, salary deductions, or penalties?		
Yes	6	5.8%
No	97	94.2%
Have you ever published scientific research or participated in a patent?		
Yes	32	31.1%
No	71	68.9%
Do you see that nepotism (bias) influences the selection of privileged people?		
Yes	88	85.4%
No	15	14.6%
Have you ever been nominated for the Excellence Allowance?		
Yes	37	35.9%
No	66	64.1%
Have you ever been nominated for the monthly certificate of excellence?		
Yes	22	21.4%
No	81	78.6%
Do you see the certificate motivating you when working?		
Yes	95	92.2%
No	8	7.8%
Is your evaluation of job performance over the last two years excellent?		
Yes	99	96.1%
No	4	3.9%
Is the team excellence system fairer than individual excellence?		
Yes	61	59.2%
No	42	40.8%

A Likert scale was used for the remaining questions, and the scale degrees were as follows: 1 for strongly disagree, 2 for disagree, 3 for neutral, 4 for agree, and 5 for strongly agree. The distribution of the answers when asked if he or she believes his or her job performance is superior to that of his or her colleagues, are listed in Table 2.

**Table 2:** Likert scale percentage among different variables

Transparent & fair	Nomination number	Increase in performance	Positive impact	Competition	Performance	Likert scale
38.8%	8.7%	12.6%	1.9%	1.9%	3.9%	Strongly disagree
9.7%	3.9%	4.9%	2.9%	2.9%	1.9%	Disagree
29.1%	17.5%	24.3%	8.7%	10.7%	24.3%	Neutral
10.7%	10.7%	10.7%	10.7%	19.4%	25.2%	Agree
11.7%	59.2%	47.6%	75.7%	65%	44.7%	Strongly agree

Table 3 shows the mean age is 33.5 years old and that 4.05 of employees consider themselves to be performing at a higher level than their colleagues. A mean of 4.43 of employees thinks that increasing the number for the excellence allowance will result in more competition. A mean of 4.55 employees responded positively to the allowance. Majority of employees with a mean of 2.47, and 2.45 disagreed that the selection process was transparent and fair, respectively. 

**Table 3:** Mean score and p-value of different variables The mean score and p-value are significant if <0.05 using a one-sample t-test for the variable "Do you see your job performance as better than your colleague's?" in comparison with the remaining variables.

		Mode	Mean	Variance		Lower Bound		*t-test p-Value
Age	101	33.50	33.50	.524		32.08		0.000
Do you see your job performance as better than your colleague's?	103	4.05	4.05	.011		3.84		0.000
Do you think increasing the number of employees involved in the allowance will increase competition?	103	4.43	4.43	.009		4.24		0.000
Do you see the excellence allowance having a positive effect on your performance?	103	4.55	4.55	.008		4.37		0.000
Did your productivity increase because of the excellence allowance?	103	3.76	3.76	.020		3.48		0.000
Do you see the nomination rate as low and does not match the employee number?	103	4.08	4.08	.017		3.82		0.000
Do you think that the selection mechanism for the excellence allowance is transparent?	103	2.47	2.47	.020		2.19		0.000
Do you think that the selection mechanism for excellence allowance is fair?	103	2.45	2.45	.017		2.19		0.000

## Discussion

This study was conducted at Aseer Central Hospital, a tertiary hospital in Abha, Saudi Arabia, to evaluate the opinions of the HCPs about the excellence allowance and its effects on their job performance. We expect that the solutions we suggest here will help overcome the difficulties HCP reported. The results of the study confirm that HCPs are in need of more education about the regulations and standards, requirements, and mechanism of selection for the excellence allowance; half the participants did not have good knowledge about them, and less than a quarter thought the selection was transparent or fair.

The absence rate in the study area was 7.8%, which reflects a high percentage of compliance from the staff. The staff previously engaged in research activity at a rate of 31.1%, which they considered acceptable. This reflects the need for a research training program and more encouragement for using the scientific method and the most recent study to solve daily healthcare issues. A very high rate of 88.4% of employees thinks there is selection bias in the nominations for the excellence program. When we look at the history of employees, we find that 35.9% have received excellence awards in the last few years. Otherwise, 21.4% of the staff receive a monthly excellence certificate. Regarding the impact of the program, 92.2% shows there is a positive impact of the program on their performance, which is compatible with the global study [19,20].

There is another suggestion from 59.2% of staff about using team excellence to replace individual excellence to encourage a cooperative teamwork environment and decrease the conflict of interest between staff and personal sensitivity [21]. Many of the staff members with a mean of 4.05 demonstrate superior performance to their colleagues, indicating a high rate of self-reported bias. As a result, there is a lower level of satisfaction, a lack of trust in transparency, and in severe cases, a negative impact on their behavior and performance.

The study's limitations are that it was conducted in only one hospital, which resulted in a lack of generalization, and that the variable was self-reporting, which resulted in a degree of self-reported bias. To be more accurate, we recommend that future studies collect performance and absenteeism feedback directly from systems and that the study be conducted in multiple hospitals to be more generalizable.

Recommendations

Educational lectures by experts, awareness-raising infographics and videos via emails, and interdepartmental and institutional meetings are suggested solutions to clarify the ambiguous points to encourage more HCPs and raise their interest. This information will definitely make more people want to get the excellence allowance, which will then motivate them to do a better job.

Also, it will reduce or even remove the widespread thoughts of favoritism some have. More than two-thirds of the participants stated that they have prior experience with either paper publication or obtaining an invention patent, indicating that they have the desire but face some challenges and are unfamiliar with such issues. Also, HCPs are more inclined to get the monthly excellence reward and get excellent grades in their yearly evaluation. This indicates getting the monthly reward and an excellent degree in the yearly evaluation is easier because the excellence allowance has more requirements, which are even more difficult. This can be overcome by attending training courses, holding conferences and meetings, and making agreements with researchers to allow some HCPs to work with them when conducting studies. When we look at the results, we see that 44.7% of the staff does not know what the criteria of an excellence program are, which means we need to educate all staff about the program goal, objectives, criteria, and strategy of the health care organization.

## Conclusions

The HCP has to realize the importance of teamwork and its positive effects, like facilitating and speeding up work and increasing productivity while reducing individual workloads. This can be achieved by informing employees of some experiences in this field, letting them practice teamwork in some situations, and thereby realizing its advantages. It will be better if the number of candidates for the excellence allowance is increased because the majority of the participants think it is low and does not match the number of employees. This will increase the opportunity to have a chance. This study demonstrates the need for increasing the knowledge of employees about the excellence program to encourage them and increase their opportunities for rewards for having a positive impact on implementing the health organization's strategy and improving healthcare delivery and quality. There is a high percentage of employees who are dissatisfied with the transparency and fairness of the selection criteria, and some of them believe that team performance should be improved rather than individual performance. So, the recommendation is to improve the selection criteria, make them more specific and transparent, and increase the number of teams and individuals who can be enrolled to improve the healthcare delivery system.
